# Long-branch attraction and the phylogeny of true water bugs (Hemiptera: Nepomorpha) as estimated from mitochondrial genomes

**DOI:** 10.1186/1471-2148-14-99

**Published:** 2014-05-07

**Authors:** Teng Li, Jimeng Hua, April M Wright, Ying Cui, Qiang Xie, Wenjun Bu, David M Hillis

**Affiliations:** 1 Institute of Entomology, College of Life Sciences, Nankai University, 94 Weijin Road, Tianjin 300071, China; 2 Department of Integrative Biology, University of Texas at Austin, Austin TX 78712, USA

**Keywords:** Long-branch attraction, Nepomorpha, Mitochondrial genome, Taxon sampling, Likelihood-ratio test

## Abstract

**Background:**

Most previous studies of morphological and molecular data have consistently supported the monophyly of the true water bugs (Hemiptera: Nepomorpha). An exception is a recent study by Hua et al. (BMC Evol Biol 9: 134, 2009) based on nine nepomorphan mitochondrial genomes. In the analysis of Hua et al. (BMC Evol Biol 9: 134, 2009), the water bugs in the group Pleoidea formed the sister group to a clade that consisted of Nepomorpha (the remaining true water bugs) + Leptopodomorpha (shore bugs) + Cimicomorpha (assassin bugs and relatives) + Pentatomomorpha (stink bugs and relatives), thereby suggesting that fully aquatic hemipterans evolved independently at least twice. Based on these results, Hua et al. (BMC Evol Biol 9: 134, 2009) elevated the Pleoidea to a new infraorder, the Plemorpha.

**Results:**

Our reanalysis suggests that the lack of support for the monophyly of the true water bugs (including Pleoidea) by Hua et al. (BMC Evol Biol 9: 134, 2009) likely resulted from inadequate taxon sampling. In particular, long-branch attraction (LBA) between the distant outgroup taxa and Pleoidea, as well as LBA among taxa in the ingroup, made Nepomorpha appear to be polyphyletic. We used three complementary strategies to test and alleviate the effects of LBA: (1) the removal of distant outgroups from the analysis; (2) the addition of closely related outgroups; and (3) the addition of a mitochondrial genome from a second family of Pleoidea. We also performed likelihood-ratio tests to examine the support for monophyly of Nepomorpha with different combinations of taxa included in the analysis. Furthermore, we found that specimens of *Helotrephes* sp. were misidentified as *Paraplea frontalis* (Fieber, 1844) by Hua et al. (BMC Evol Biol 9: 134, 2009).

**Conclusions:**

All analyses that included the addition of more taxa significantly and consistently supported the placement of Pleoidea within the Nepomorpha (i.e., supported the monophyly of the traditional true water bugs). Our analyses further support a close relationship between Notonectoidea and Pleoidea within Nepomorpha, and the superfamilies Nepoidea, Ochteroidea, Naucoroidea, and Pleoidea are resolved as monophyletic in all trees with strong support. Our results also confirmed that monophyly of Nepomorpha clearly is not refuted by the mitochondrial genome data.

## Background

Long-branch attraction (LBA) is a bias that results in spurious support for relationships between two (or more) long branches in an estimated phylogenetic tree when the assumed model of evolution is too simplistic [1,2]. Biases associated with LBA have been identified in many phylogenetic studies, including analyses of mammals [3,4], birds [5], arthropods [6-8], and seed plants [9,10]. The most common problem occurs when distantly related ingroup taxa are poorly sampled and one or a few distant outgroup taxa are included to root the tree. Under these conditions, a simplistic model of evolution is unlikely to sufficiently account for homoplasy, and long branches will be connected (or attracted to one another) in the inferred tree based on homoplastic similarities [11]. One method for detecting this problem involves conducting phylogenetic analyses with and without outgroups [12]. If the inclusion of a distant outgroup changes the inferred relationships of the ingroup, it may be better to infer ingroup relationships separately and consider other methods for rooting the resulting tree, or to use more closely related outgroups [13]. In addition, several strategies have been suggested to reduce the effects of LBA, including: (1) excluding long-branch taxa from the analysis, (2) replacing the long-branch taxa with slow-evolving close relatives, (3) removing fast-evolving proteins or sites, (4) improving the models of character evolution assumed in the analysis, and (5) sampling more taxa to break up long branches in the tree [14-16]. Among these methods, adding taxa to break up long branches is one of the most widely suggested strategies to reduce the effects of LBA bias [17,18]. Appropriate and thorough taxon sampling is thus one of the most important considerations for accurate phylogenetic estimation [16-19]. Phylogenetic analyses based on relatively few distantly related taxa (but with each taxon represented by many characters, such as from a mitochondrial genome) are particularly prone to problems with LBA; such analyses are likely to produce high support values for incorrect phylogenetic relationships [16,20].

**Figure 1:**
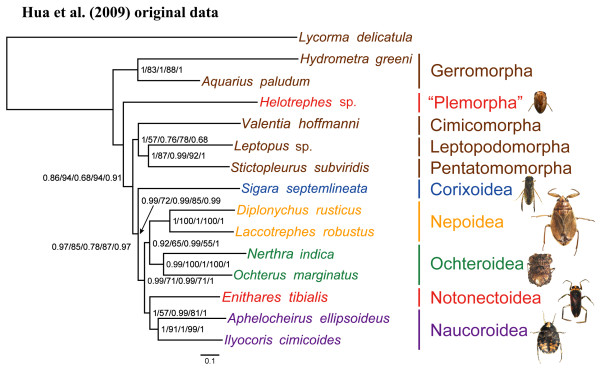
**The consensus phylogeny based on the data sets analyzed by Hua et al. [**[28]**].** Five of the eight phylogenetic analyses they conducted supported this tree. Numbers at the nodes indicate the BPP and ML support values for each data matrix analyzed by Hua et al. [28] in the following order: PP and BP for PCG123RT, PP and BP for PCG12RT, and PP for PCG12. Branch lengths are similar across analyses; these branch lengths represent the analysis of the PCG123RT data set. The scale bar represents the number of expected substitutions per site.

The relationships of the true water bugs (Hemiptera: Nepomorpha) within heteropteran insects [21] have been the subject of many studies of molecular and morphological data. The monophyly of Nepomorpha has been consistently and strongly supported by studies based on morphological characters [22-25], molecular data (partial sequences of 16S rDNA and 28S rDNA [26], and four Hox genes [27]), and by combined data analyses [26]. In contrast, the monophyly of Nepomorpha has only been disputed in the study of Hua et al. [28], who based their analysis on nine nepomorphan mitochondrial genomes (mt-genomes). In the study by Hua et al. [28], Pleoidea was not supported as part of Nepomorpha, but instead was resolved as the sister-group of a clade that included the remaining species of Nepomorpha plus Leptopodomorpha, Cimicomorpha, and Pentatomomorpha (Figure 1). As a result of these analyses, Hua et al. [28] suggested that Pleoidea should be raised from a superfamily within Nepomorpha to the infraorder Plemorpha, outside of Nepomorpha. Their conclusions were supported by high Bayesian posterior probabilities (BPP) and maximum likelihood (ML) bootstrap proportions in five of eight phylogenetic analyses.

The study by Hua et al. [28] has both strengths and weaknesses when compared with previous studies of the phylogenetic relationships of Nepomorpha. Each taxon sampled by Hua et al. [28] was sampled for complete mitochondrial genomes, so the number of characters available for phylogenetic inference was large. In contrast, previous studies [22-27] examined fewer characters per taxon, but included more taxa in the analyses. Thorough taxon sampling can often lead to more accurate phylogenetic inference, even if the total number of characters in the analysis is decreased [29-32]. In particular, the position of Pleoidea in the study of Hua et al. [28] may have been affected by the inclusion of just one of two families in Pleoidea (Helotrephidae, without any representation of Pleidae; see Results and discussion). This made it more likely for the tree to be rooted by connection of the distantly related outgroup taxa to the long branch leading to *Helotrephes* sp. (Figure 1).

A second consideration is the selection of outgroups used by Hua et al. [28]. Fulgoromorpha is very distantly related to the ingroup Nepomorpha, making problems associated with LBA more likely [30,33]. Furthermore, in groups more closely related to Nepomorpha, Hua et al. [28] sampled only one representative for three different infraorders (Cimicomorpha, Leptopodomorpha and Pentatomomorpha). Thus, we examined the possibility that the findings of Hua et al. [28] resulted from biases associated with inadequate taxon sampling. Because the model-based methods used by Hua et al. [28] are less sensitive to the problems of LBA [34-36], these authors did not consider LBA to be a likely explanation of their results. However, models of evolution are never perfect, and poor taxon sampling exacerbates the problems of model insufficiency, so the use of model-based inference methods is not, by itself, a panacea for dealing with biases associated with LBA [11,16].

We undertook the current study to explore the conclusion of Hua et al. [28] that the Pleoidea evolved their fully aquatic lifestyle independently of the remaining true water bugs in Nepomorpha. Our hypothesis was that this conclusion was a result of LBA between the single sampled representative of Pleoidea and the distantly related outgroup, Fulgoromorpha. We tested this hypothesis by: (1) removing the outgroups and re-estimating the phylogeny of Nepomorpha only, to detect whether the ingroup topology is affected by the long-branch outgroup taxa [12,13]; (2) increasing taxon sampling of groups related to Nepomorpha, including Leptopodomorpha, Cimicomorpha, and Pentatomomorpha [37]; and (3) adding new mt-genome data for a representative of the second family within Pleoidea, namely Pleidae (the presumed sister-group of Helotrephidae).

## Results and discussion

### Misidentification of previously sampled taxa

To test our hypothesis that the conclusion of Hua et al. [28] (Pleidae outside of the remaining Nepomorpha) was an artifact of limited taxon sampling, we sampled a member of the family Helotrephidae. Helotrephidae is generally accepted as the sister-group of Pleidae [22,23,25,26], so we reasoned that including the sister-group of Pleidae was the best way to break up the long terminal branch leading to this taxon. We sequenced the mt-genome of *Helotrephes semiglobosus semiglobosus* Stål, 1860 (Nepomorpha: Helotrephidae). However, after we obtained a partial mt-genome sequence of *Helotrephes semiglobosus semiglobosus* (GenBank accession number: KJ027513) with the length of 8,876 bp, including 29 genes (two rRNAs, ten protein coding genes [PCGs] and 17 tRNAs) as well as the control region, we found extreme similarity (97.4%) between this species and the specimen previously identified by Hua et al. [28] as *Paraplea frontalis* (Fieber, 1844). As this level of sequence similarity was unexpected between species in these two families, we checked the specimens identified previously as *Paraplea frontalis* by Hua et al. [28]. We found that those specimens are properly identified as *Helotrephes* sp., and so represent a species in Helotrephidae rather than Pleidae. As the mt-genome of a species in Helotrephidae was already represented in the data set, we then sequenced a new mt-genome of *Paraplea frontalis*, as a true representative of Pleidae. Henceforth, we label the sample sequenced by Hua et al. [28] correctly as *Helotrephes* sp..

### Removal of outgroups from the analysis

**Figure 2:**
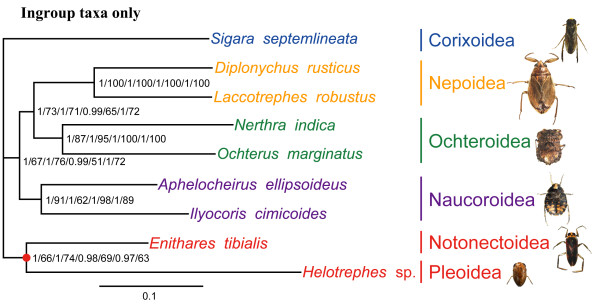
**Phylogenetic results based on analyses of ingroup taxa only.** Numbers at the nodes are BPP and ML support values in the following order: PP and BP for PCG12, PP and BP for PCG123, PP and BP for PCG12RT, and PP and BP for PCG123RT. The red dot on the tree indicates the clade of Notonectoidea + Pleoidea. The scale bar represents the number of expected substitutions per site based on analysis of the PCG12 data set.

The most common problem of LBA is that distantly related outgroups have a biased attraction to long branches within the ingroup [3,4,38]. For this reason, a common suggestion is to conduct phylogenetic analyses both with and without the outgroups to compare whether the distantly related outgroup alters the ingroup topology [16]. To test if outgroup selection affected the topology of our ingroup, we ran analyses using only the ingroup taxa of Hua et al. [28]. Using Bayesian and ML analyses, all data matrices of Hua et al. [28] generated phylogenetic trees with the same topology (Figure 2). When the outgroups are removed, the ingroup topology is distinct from that obtained by Hua et al. [28] (Figure 1). In all of these analyses, *Helotrephes* sp. was connected to *Enithares tibialis* Liu et Zheng, 1991 (Nepomorpha: Notonectoidea).

### Addition of outgroups

Outgroup selection is an important factor for reconstructing phylogenetic trees, because the choice of outgroup taxa can affect the ingroup topology [39]. However, outgroup selection is often not adequately considered [40,41]. Moreover, several authors have pointed out that adding more outgroup taxa in the sister-group to a phylogenetic analysis can improve the accuracy of phylogenetic estimation, and also should help break up the LBA between any long-branch members of the ingroup and the outgroup [38,42,43]. Therefore, we added three more taxa (selected from the sister-group of Nepomorpha) to the dataset of Hua et al. [28].

**Figure 3:**
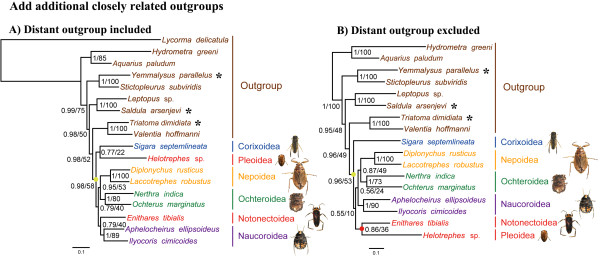
**Phylogenetic trees based on the inclusion of additional closely related outgroups. (A)** Analysis including the distant outgroup *Lycorma delicatula* (Hemiptera: Auchenorrhyncha: Fulgoromorpha). **(B)** Analysis excluding the distant outgroup *Lycorma delicatula*. Numbers at the nodes are BPP (left) and ML support values (right). Yellow dots on each phylogram indicate the clades of Nepomorpha, and red dot indicate the clades of Notonectoidea + Pleoidea. Asterisks indicate these additional closely related outgroups. The scale bar represents the number of expected substitutions per site.

Both Bayesian inference and ML analyses resulted in the same topology (Figure 3A); the position of the long branch of *Helotrephes* sp. (Nepomorpha: Pleoidea) was supported within Nepomorpha rather than outside of Nepomorpha, in contrast to the findings of Hua et al. [28]. The monophyly of Nepomorpha (including both Helotrephidae and Pleidae) received strong support in Bayesian analyses (based on posterior probabilities: PP) but with relatively weak support in ML analyses (based on bootstrap proportions: BP). The monophyletic Nepoidea, Ochteroidea, and Naucoroidea were strongly supported by both PP and BP, similar to the results of Hua et al. [28]. Additionally, the topology of the infraordinal relationships of Heteroptera is similar to previous work [44] also based on mt-genomes, namely (Gerromorpha + (Pentatomomorpha + (Leptopodomorpha + (Cimicomorpha + Nepomorpha)))).

We also estimated phylogenetic trees without the long-branched outgroup of *Lycorma delicatula* (White, 1845) (Hemiptera: Auchenorrhyncha: Fulgoromorpha). The major changes that resulted from deletion of this taxon were the position of *Helotrephes* sp. and Naucoroidea (Figure 3B). In both Bayesian and ML analyses, *Helotrephes* sp. (Nepomorpha: Pleoidea) was supported as the sister group of *Enithares tibialis* (Nepomorpha: Notonectoidea). The close relationship between the Notonectoidea and Pleoidea also has been supported in most previous studies [22-26]. Although the relationships among families of Nepomorpha varied among trees, all the analyses that excluded Fulgoromorpha supported the monophyly of Nepomorpha (including Helotrephidae as well as Pleidae, when the latter was added to the analyses). These analyses demonstrate that the conclusions of Hua et al. [28] were at least partly a result of their use of a very distant outgroup.

### Addition of a new mitochondrial genome of Pleidae

We sequenced and assembled a new mt-genome for *Paraplea frontalis* (Fieber, 1844), except for small portions of 12S rRNA gene and the control region (polynucleotide sequences in these two regions proved difficult to resolve with certainty). This mt-genome was 14,143 bp in length and has been deposited in the GenBank (Accession number: KJ027516). The mt-genome of *Paraplea frontalis* contained the typical 37 genes (two rRNAs, 13 PCGs and 22 tRNAs), with the same gene order as observed in most other true bugs [44,45] (Table 1). Gene overlaps were found at 11 gene junctions and involved a total of 32 bp, which may make the genome relatively compact. Twelve of the 13 PCGs initiated with ATN as start codon, whereas the COI gene started with TTG. Eight PCGs ended with the termination codon TAA and one with TAG, whereas the remaining four were terminated with T. All of the 22 typical animal tRNA genes were observed in the *Paraplea frontalis* mt-genome, ranging from 63 to 74 bp. Most of the tRNAs could be folded into typical cloverleaf secondary structures, except that the stem of the dihydrouridine (DHU) arm simply formed a loop in tRNA-Ser (GCT) (see Additional file 1). There are 22 unmatched base pairs in the *Paraplea frontalis* mitochondrial tRNA secondary structures.

**Table 1:** **Organization of the *Paraplea frontalis *** **mitochondrial genome**

**Gene**	**Strand**	**Position**	**Anticodon**	**Size (bp)**	**Start codon**	**Stop codon**	**Intergenic nucleotides**^**a**^
tRNA-Ile	J	1-64	GAT	64			
tRNA-Gln	N	62-130	TTG	69			-3
tRNA-Met	J	131-199	CAT	69			0
ND2	J	200-1201		1002	ATT	TAA	0
tRNA-Trp	J	1203-1268	TCA	66			1
tRNA-Cys	N	1261-1324	GCA	64			-8
tRNA-Tyr	N	1325-1391	GTA	67			0
COI	J	1393-2931		1539	TTG	TAA	1
tRNA-Leu	J	2927-2991	TAA	65			-5
COII	J	2992-3670		679	ATA	T-	0
tRNA-Lys	J	3671-3744	CTT	74			0
tRNA-Asp	J	3744-3806	GTC	63			-1
ATPase8	J	3807-3962		156	ATA	TAA	0
ATPase6	J	3956-4666		667	ATG	TAG	-7
COIII	J	4623-5409		787	ATG	T-	-44
tRNA-Gly	J	5410-5472	TCC	63			0
ND3	J	5473-5826		354	ATA	TAA	0
tRNA-Ala	J	5850-5913	TGC	64			23
tRNA-Arg	J	5914-5979	TCG	66			0
tRNA-Asn	J	5979-6044	GTT	66			-1
tRNA-Ser	J	6044-6113	GCT	70			-1
tRNA-Glu	J	6114-6178	TTC	65			0
tRNA-Phe	N	6177-6242	GAA	66			-2
ND5	N	6243-7944		1702	ATG	T-	0
tRNA-His	N	7946-8009	GTG	64			1
ND4	N	8009-9346		1338	ATG	TAA	-1
ND4L	N	9349-9651		303	TTG	TAA	2
tRNA-Thr	J	9654-9717	TGT	64			2
tRNA-Pro	N	9718-9782	TGG	65			0
ND6	J	9785-10285		501	ATT	TAA	2
CytB	J	10285-11421		1137	ATG	TAG	-1
tRNA-Ser	J	11420-11487	TGA	68			-2
ND1	N	11504-12421		918	ATT	TAA	16
tRNA-Leu	N	12422-12485	TAG	64			0
16S rRNA	N	12486-13757		1272			0
tRNA-Val	N	13758-13827	TAC	70			0
12S rRNA	N	13828-14143		316			0

^a^Numbers correspond to nucleotides separating a gene from an upstream one; negative numbers indicate that adjacent genes overlap.

**Figure 4:**
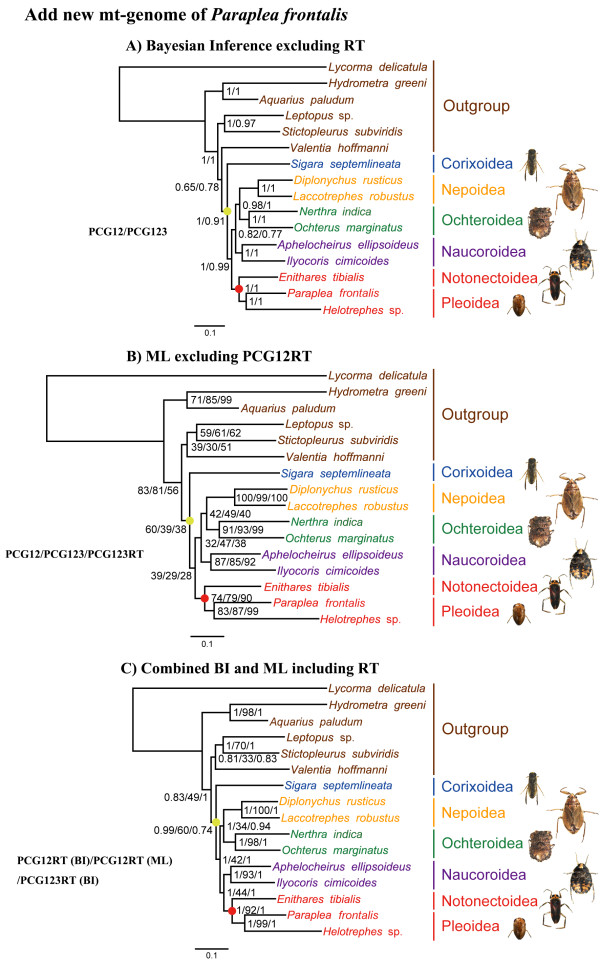
**Phylogenetic trees based on the addition of a new mitochondrial genome of *Paraplea frontalis *(Nepomorpha: Pleoidea).** With adding the new mt-genome of *Paraplea frontalis* (Fieber, 1844) to the data matrices of Hua et al. [28], we gathered four new data matrices of 16(PCG12), 16(PCG123), 16(PCG12RT), and 16(PCG123RT). **(A)** Numbers at the nodes are BPP for the data matrix of 16(PCG12) (left) and 16(PCG123) (right). **(B)** Numbers at the nodes are ML support values for the data matrix of 16(PCG12) (left), 16(PCG123) (middle), and 16(PCG123RT) (right). **(C)** Numbers at the nodes are BPP for 16(PCG12RT) (left), ML support values for 16(PCG12RT) (middle), and BPP for 16(PCG123RT) (right). Yellow dots on each phylogram indicate the clades of Nepomorpha, and Red dots indicate the clades of Notonectoidea + Pleoidea. The scale bar represents the number of expected substitutions per site.

Increased taxon sampling, especially when it breaks up long branches in a tree, is the most effective strategy for reducing the effects of LBA [16,31,32]. We added the representative of Pleidae, which is thought to be the sister-group of Helotrophidae, to help reduce the length of the branch that led to the single sampled species of Helotrephidae sampled by Hua et al. [28]. We therefore added our mt-genome of *Paraplea frontalis* to the four data matrices of Hua et al. [28] and conducted new phylogenetic analyses (Figure 4).

As with our analyses that replaced the distant outgroup with more appropriate outgroups, the analyses that included a member of Pleidae supported monophyly of Nepomorpha (with strong PP support but weak BP support). Moreover, these analyses strongly supported *Paraplea frontalis* (Pleidae) as the sister group of *Helotrephes* sp. (Helotrephidae). Together, Pleidae and Helotrephidae were supported as the sister-group of Notonectidae. The monophyletic groups of Nepoidea, Ochteroidea, Naucoroidea, Pleoidea, and Notonectoidea + Pleoidea were strongly supported by both PP and BP in all analyses that included Pleidae.

### Likelihood-ratio tests

We compared the likelihood ratios of the best solutions for each of our two alternative hypotheses (Pleoidea inside versus outside of Nepomorpha; see Additional file 2) for eight different combinations of taxa (Table 2). The monophyly of Nepomorpha (including Pleoidea) was strongly supported if we added *Paraplea frontalis* and/or three more outgroup taxa to the original data matrix of Hua et al. [28], as well as when we analyzed the data set without the distant outgroup consisting of *Lycorma delicatula*. The original conclusion of Hua et al. [28] (the polyphyly of true water bugs) was only supported with the specific combination of taxa analyzed in the original study. Even then, the likelihood-ratio support for this result over the alternative is weak (Table 2).

**Table 2:** Likelihood-ratio tests for monophyly of Nepomorpha with eight different combinations of taxa

**Taxa added to analysis of Hua et al. **[28]	**Taxa deleted from analysis of Hua et al. **[28]	**ln L (Hypothesis 1)**	**ln L (Hypothesis 2)**	**2ΔL**	**Hypothesis 1**^***a ***^**(Helotrephidae within Nepomorpha)**	**Hypothesis 2**^***a ***^**(Helotrephidae outside Nepomorpha)**
None	None	-68913.45	-68909.21	8.48		Weak
None	*Lycorma delicatula*	-63056.47	-63052.68	7.58		Weak
*Paraplea frontalis*	None	-72517.55	-72552.52	-69.94	Very strong	
*Paraplea frontalis*	*Lycorma delicatula*	-66486.5	-66522.49	-71.98	Very strong	
*Triatoma dimidiata*	None	-80054.05	-80074.67	-41.24	Very strong	
*Yemmalysus parallelus*						
*Saldula arsenjevi*						
*Triatoma dimidiata*	*Lycorma delicatula*	-74196.98	-74230.9	-67.84	Very strong	
*Yemmalysus parallelus*						
*Saldula arsenjevi*						
*Paraplea frontalis*	None	-83521.55	-83612.5	-181.9	Very strong	
*Triatoma dimidiata*						
*Yemmalysus parallelus*						
*Saldula arsenjevi*						
*Paraplea frontalis*	*Lycorma delicatula*	-77682.65	-77774.22	-183.14	Very strong	
*Triatoma dimidiata*						
*Yemmalysus parallelus*						
*Saldula arsenjevi*						

*a*: 2ΔL scores with an absolute value of 0 to 10 indicate weak support, >10 to 30 indicate strong support, and >30 indicate very strong support for the favored hypothesis [48]. Scores are calculated so that positive values indicate support for Hypothesis 2, and negative values indicate support for Hypothesis 1.

### Phylogeny of nepomorpha

Given that the monophyly of Nepomorpha is consistently supported in all of our new analyses, we find no support for the new infraorder Plemorpha. Therefore, we recommend retaining Pleoidea as part of Nepomorpha. The superfamilies of Nepoidea (Belostomatidae + Nepidae), Ochteroidea (Gelastocoridae + Ochteridae), Naucoroidea (Aphelocheiridae + Naucoridae), and Pleoidea (Pleidae + Helotrephidae) are monophyletic groups in all our analyses with high support from both PP and BP. We also found strong support for the close relationship between Notonectoidea and Pleoidea. Several synapomorphies of biological and ecological traits also support some of these monophyletic groups [24-26,46]:

Nepomorpha: the short antennae are concealed below the eyes; all have an aquatic lifestyle, although Ochteroidea (including Ochteridae and Gelastocoridae) live along freshwater shores rather than underwater;

Nepoidea (including Nepidae and Belostomatidae): air-breathing through a siphon;

Naucoroidea: all Aphelocheiridae and some Naucoridae use plastron respiration;

Pleoidea (including Pleidae and Helotrephidae): also have plastron respiration, which allows them to stay permanently submerged;

Notonectoidea and Pleoidea (including Notonectidae, Pleidae, and Helotrephidae): swim on their backs in an inverted position.

Our principal goal in this study was to discuss the monophyly of Nepomorpha and the effects of adequate taxon sampling on this phylogenetic problem. As we did not sample all the families of Nepomorpha, a more thorough sampling of taxa is needed to adequately resolve the family relationships within Nepomorpha. In particular, more sampling of Potamocoridae, Micronectidae and Diaprepocoridae (Hemiptera: Nepomorpha) mt-genome sequences will be needed for a thorough analysis of the major groups within Nepomorpha.

## Conclusions

This study provides a clear example of the importance of adequate sampling. We support the conclusion that investigators should be cautious about making major taxonomic rearrangements on the basis of limited taxon sampling, even (or especially) when the number of characters sampled per taxon is large [16,17,31,32]. Phylogenetic analyses that are based on even complete genomes of relatively few taxa are likely to result in strongly supported, but incorrect, evolutionary reconstructions [16,17,47]. In the study by Hua et al. [28], limited sampling of mt-genomes, coupled with the use of a distant outgroup, resulted in a conclusion that was at odds with a traditionally supported group (true water bugs, or Neopmorpha). But even minimal additional sampling to break up long branches in the tree, or the use of more closely related outgroups, results in trees in which the traditional group Nepomorpha is supported.

In the phylogenomic era [48], many papers are reporting surprising phylogenetic results that conflict with traditional hypotheses of relationships. Many (or even most) of these surprising results are based on analyses of many characters (even whole genomes) from very few taxa [16,47,49]. Strong “statistical support” for a given conclusion may come from strong underlying phylogenetic signal, but also from systematic bias that stems from assuming inadequate or inappropriate models of evolution [50]. Using large numbers of characters in a phylogenetic analysis means that even small systematic biases associated with overly simplistic methodological assumptions are likely to be mistaken as strong phylogenetic signal. Thorough taxon sampling allows the use of more simplistic models of evolution, because multiple changes at each nucleotide site can be appropriately reconstructed through the increased sampling of the tree [18]. If the sampling in a phylogenomic study is sparse, investigators should use appropriate caution before overturning analyses that are based on more thorough sampling of taxa.

## Methods

### Ethics statement

No specific permits were required for the insect collected for this study in Yunnan and Hubei Province, China. The insect specimens were collected with a sturdy aquatic net at the pond. The field studies did not involve endangered or protected species. The species in the genus of *Paraplea* and *Helotrephes* are common small insects and are not included in the “List of Protected Animals in China”.

### Specimen collection

Adult specimens of *Paraplea frontalis* were collected from Tongbiguan Village (24°36.411 N, 97°39.349E), Yingjiang County, Dehong City, Yunnan Province, China, on May 18th, 2009. Adult specimens of *Helotrephes semiglobosus semiglobosus* were collected from Jin Ji Valley (29°22.339 N, 114°34.301E), Jiu Gong Shan, Tong Shan County, Hubei Province, China, on July 30th, 2010. Voucher specimens are deposited in the Insect Molecular Systematics Lab, Institute of Entomology, College of Life Sciences, Nankai University, Tianjin, China. All specimens were initially preserved in 95% ethanol in the field. After being transferred to the laboratory, they were stored at -20°C until used for DNA extraction.

### PCR amplification and sequencing

Whole genomic DNA was extracted from thoracic muscle tissue by CTAB-based method [51]. The mt-genome of *Paraplea frontalis* was amplified in four overlapping PCR fragments by PCR amplification (see Additional file 3). The partial mt-genome of *Helotrephes semiglobosus semiglobosus* was sequenced with two fragments (see Additional file 4). Primer pairs were modified from previous work [28], and designed from sequenced fragments.

PCR reactions were performed with TaKaRa LA *Taq* under the following conditions: 1 min initial denaturation at 94°C, followed by 30 cycles of 20 s at 94°C, 1 min at 50°C, and 2–8 min at 68°C, and a final elongation for 10 min at 72°C. PCR products were electrophoresed in 1% agarose gel, purified, and then sequenced using an ABI 3730XL capillary sequencer with the BigDye Terminator Sequencing Kit (Applied Bio Systems). All fragments were sequenced with primer walking on both strands.

### Sequence analysis and annotation

Sequence files were assembled into contigs using BioEdit version 7.0.5.2 [52]. Protein coding regions were determined via ORF Finder implemented at the NCBI website (http://www.ncbi.nlm.nih.gov/gorf/gorf.html) with invertebrate mitochondrial genetic codes. Transfer RNA analysis was performed by tRNAscan-SE version 1.21 [53] with the invertebrate mitochondrial codon predictors and a cove score cut-off of 5. Few tRNA genes that could not be identified by tRNAscan-SE were determined by comparing to other heteropterans. Analyses of sequences were performed with MEGA version 5.0 [54].

### Taxon sampling

In total, 19 taxa were sampled. These taxa included representatives of 10 out of 11 extant families of Nepomorpha [46,55] and 9 outgroups (Table 3). Among them, the mt-genome data of *Paraplea frontalis* is reported here for the first time. To make the results more directly comparable to the study of Hua et al. [28], we retrieved all mt-genomes of 15 taxa (including nine ingroups and six outgroups) from their work. According to the analysis of the heteropteran infraorders of Wheeler et al. [37], the phylogenetic relationships of Heteroptera are as follows: (Enicocephalomorpha + (Dipsocoromorpha + (Gerromorpha + (Nepomorpha + (Leptopodomorpha + (Cimicomorpha + Pentatomomorpha)))))). Therefore, we sampled another three taxa within the sister group to Nepomorpha as outgroups, with one representative from each of Leptopodomorpha, Cimicomorpha and Pentatomomorpha.

**Table 3:** Taxonomy and GenBank accession numbers of mitochondrial genomes for species sampled in this study

**Suborder (bold) Infraorder (not bold)**	**Superfamily**	**Family**	**Species**	**Accession number**
**Auchenorrhyncha**				
Fulgoromorpha	Fulgoroidea	Fulgoridae	*Lycorma delicatula*	NC_012835
**Heteroptera**				
Gerromorpha	Hydrometroidea	Hydrometridae	*Hydrometra greeni*	NC_012842
	Gerroidea	Gerridae	*Aquarius paludum*	NC_012841
Leptopodomorpha	Saldoidea	Saldidae	*Saldula arsenjevi*	NC_012463
	Leptopodoidea	Leptopodidae	*Leptopus* sp.	FJ456946
Cimicomorpha	Reduvioidea	Reduviidae	*Triatoma dimidiata*	NC_002609
	Reduvioidea	Reduviidae	*Valentia hoffmanni*	NC_012823
Pentatomomorpha	Lygaeoidea	Berytidae	*Yemmalysus parallelus*	NC_012464
	Coreoidea	Rhopalidae	*Stictopleurus subviridis*	NC_012888
Nepomorpha	Corixoidea	Corixidae	*Sigara septemlineata*	FJ456941
	Nepoidea	Belostomatidae	*Diplonychus rusticus*	FJ456940
		Nepidae	*Laccotrephes robustus*	FJ456948
	Ochteroidea	Gelastocoridae	*Nerthra indica*	FJ456943
		Ochteridae	*Ochterus marginatus*	FJ456950
	Naucoroidea	Naucoridae	*Ilyocoris cimicoides*	NC_012845
		Aphelocheiridae	*Aphelocheirus ellipsoideus*	FJ456939
	Notonectoidea	Notonectidae	*Enithares tibialis*	NC_012819
	Pleoidea	Helotrephidae	*Helotrephes* sp.	FJ456951
		Pleidae	*Paraplea frontalis*	KJ027516

### Phylogenetic analyses

All PCGs were aligned based on their amino acid sequences using MUSCLE as implemented in the MEGA version 5.0 [54]. The rRNAs and tRNAs were aligned with CLUSTAL_X version 1.83 [56] under the default settings. The alignments of tRNA genes were corrected according to the secondary structures, especially the stem regions. The aligned nucleotide sequences, excluding stop codons, were then concatenated and used to reconstruct the phylogeny. All phylogenetic trees were built using only first and second codon positions of 13 PCGs, except in our analyses in which we removed or added taxa to the data matrices of Hua et al. [28], so that we could make a direct comparison using methods used in the original paper. Our analyses with added and deleted taxa used the same data sampling methods of Hua et al. [28]; these analyses contained four kinds of data matrices: (1) The PCG123RT matrix, including all three codon positions of PCGs, rRNA genes, and tRNA genes; (2) the PCG12RT matrix, including the first and the second codon positions of PCGs, rRNA genes, and tRNA genes; (3) the PCG123 matrix, including all the three codon positions of PCGs; and (4) the PCG12 matrix, including the first and the second codon positions of PCGs.

We used GPU MrBayes [57] for Bayesian inference and raxmlGUI 1.2 [58] for ML analyses to reconstruct phylogenetic trees. We used the GTR + I + Γ model, based on results from Modeltest Version 3.7 [59]. In Bayesian inference, two simultaneous runs of 10,000,000 generations were conducted for each matrix. Each set was sampled every 100 generations. Trees that were sampled prior to stationarity (at 25% of the run) were discarded as burnin, and the remaining trees were used to construct a 50% majority-rule consensus tree. For the ML analysis, we conducted 1000 bootstrap replicates with thorough ML search.

### Tests of monophyly

Traditionally recognized taxonomic groups are usually challenged when there is strong statistical support for an alternative phylogeny [16,60]. Likelihood-ratio tests [61] can provide a powerful means of examining alternatives. We applied likelihood-ratio tests to compare the support of various data sets for two different hypotheses (see Additional file 2):

Hypothesis 1: Helotrephidae is nested within Nepomorpha (i.e., the true water bugs are monophyletic, and Helotrephidae is nested within the group).

Hypothesis 2: Helotrephidae is outside of the remaining species of Nepomorpha (i.e., true water bugs are only monophyletic if Helotrephidae is excluded from the group).

We conducted likelihood-ratio tests [61] of these two hypotheses for the original data set of Hua et al. [28], as well as with various additions and deletions of taxa, including both ingroups and outgroups. The likelihood-ratio tests were conducted using PAUP* 4 [62]. Heuristic searches were performed using the GTR + I + Γ model with 100 random addition replicates.

## Availability of supporting data

The data sets supporting the results of this article are available in the Dryad repository, http://dx.doi.org/10.5061/dryad.tf25c[63].

## Abbreviations

LBA: Long-branch attraction; Mt-genomes: Mitochondrial genomes; BPP: Bayesian posterior probabilities; ML: Maximum likelihood; PCGs: Protein coding genes; PP: Posterior probabilities; BP: Bootstrap proportions.

## Competing interests

The authors have declared that no competing interests.

## Authors’ contributions

TL designed the experiments, carried out the phylogenetic analyses, made all figures and drafted the manuscript. AMW and YC participated in the data analyses. JH and QX helped draft the manuscript. WB and DMH directed this study, designed and reviewed analyses, and revised the manuscript. All authors read and approved the final manuscript.

## Supplementary Material

Additional file 1 **Putative secondary structure of the 22 tRNAs identified in the mitochondrial genome of *Paraplea frontalis*.** The tRNAs are labeled with the abbreviations of their corresponding amino acids. Dashes indicate Watson-Crick base pairing and asterisks indicate G-U base pairing. Click here for file

Additional file 2 Constraints for the two hypotheses used in the likelihood-ratio test regarding the monophyly of Nepomorpha. Click here for file

Additional file 3 **Primers designed for *Paraplea frontalis* in this study.** Click here for file

Additional file 4 **Primers designed for *Helotrephes semiglobosus semiglobosus* in this study.** Click here for file

## References

[B1] Felsenstein J Cases in which parsimony or compatibility methods will be positively misleading Syst Zool 1978 27 4 401 410 10.2307/2412923

[B2] Hendy MD Penny D A framework for the quantitative study of evolutionary trees Syst Zool 1989 38 4 297 309 10.2307/2992396

[B3] Sullivan J Swofford DL Are guinea pigs rodents? The importance of adequate models in molecular phylogenetics J Mamm Evol 1997 4 2 77 86 10.1023/A:1027314112438

[B4] Lin YH McLenachan PA Gore AR Phillips MJ Ota R Hendy MD Penny D Four new mitochondrial genomes and the increased stability of evolutionary trees of mammals from improved taxon sampling Mol Biol Evol 2002 19 12 2060 2070 10.1093/oxfordjournals.molbev.a004031 12446798

[B5] Garcia-Moreno J Sorenson MD Mindell DP Congruent avian phylogenies inferred from mitochondrial and nuclear DNA sequences J Mol Evol 2003 57 1 27 37 10.1007/s00239-002-2443-9 12962303

[B6] Delsuc F Phillips MJ Penny D Comment on “Hexapod origins: monophyletic or paraphyletic?” Science 2003 301 5639 1482 12970547 10.1126/science.1086558

[B7] Chen W-J Bu Y Carapelli A Dallai R Li S Yin W-Y Luan Y-X The mitochondrial genome of Sinentomon erythranum (Arthropoda: Hexapoda: Protura): an example of highly divergent evolution BMC Evol Biol 2011 11 1 246 10.1186/1471-2148-11-246 21871115 PMC3176236

[B8] Schwarz MP Tierney SM Cooper SJB Bull NJ Molecular phylogenetics of the allodapine bee genus Braunsapis: A–T bias and heterogeneous substitution parameters Mol Phylogenet Evol 2004 32 1 110 122 10.1016/j.ympev.2003.11.017 15186801

[B9] Sanderson MJ Wojciechowski MF Hu JM Khan TS Brady SG Error, bias, and long-branch attraction in data for two chloroplast photosystem genes in seed plants Mol Biol Evol 2000 17 5 782 797 10.1093/oxfordjournals.molbev.a026357 10779539

[B10] Zhong B Yonezawa T Zhong Y Hasegawa M The position of Gnetales among seed plants: overcoming pitfalls of chloroplast phylogenomics Mol Biol Evol 2010 27 12 2855 2863 10.1093/molbev/msq170 20601411

[B11] Huelsenbeck JP Hillis DM Success of phylogenetic methods in the 4-taxon case Syst Biol 1993 42 3 247 264 10.1093/sysbio/42.3.247

[B12] Bergsten J A review of long-branch attraction Cladistics 2005 21 2 163 193 10.1111/j.1096-0031.2005.00059.x 34892859

[B13] Holland BR Penny D Hendy MD Outgroup misplacement and phylogenetic inaccuracy under a molecular clock–a simulation study Syst Biol 2003 52 2 229 238 10.1080/10635150390192771 12746148

[B14] Lartillot N Brinkmann H Philippe H Suppression of long-branch attraction artefacts in the animal phylogeny using a site-heterogeneous model BMC Evol Biol 2007 7 Suppl 1 S4 10.1186/1471-2148-7-S1-S4 17288577 PMC1796613

[B15] Li YW Yu L Zhang YP “Long-branch Attraction” artifact in phylogenetic reconstruction Hereditas(Beijing) 2007 29 6 659 667 10.1360/yc-007-0659 17650481

[B16] Heath TA Hedtke SM Hillis DM Taxon sampling and the accuracy of phylogenetic analyses J Syst Evol 2008 46 3 239 257

[B17] Hedtke SM Townsend TM Hillis DM Resolution of phylogenetic conflict in large data sets by increased taxon sampling Syst Biol 2006 55 3 522 529 10.1080/10635150600697358 16861214

[B18] Hillis DM Inferring complex phylogenies Nature 1996 383 6596 130 131 10.1038/383130a0 8774876

[B19] Nabhan AR Sarkar IN The impact of taxon sampling on phylogenetic inference: a review of two decades of controversy Brief Bioinform 2012 13 1 122 134 10.1093/bib/bbr014 21436145 PMC3251835

[B20] Hall BG Salipante SJ Measures of clade confidence do not correlate with accuracy of phylogenetic trees Plos Comput Biol 2007 3 3 e51 10.1371/journal.pcbi.0030051 17367204 PMC1828704

[B21] Štys P Kerzhner I The rank and nomenclature of higher taxa in recent Heteroptera Acta Entomol Bohemoslov 1975 72 2 65 79

[B22] Popov YA Historical development of the hemipterous infraorder Nepomorpha Trudy Paleontological Institute Academy of Science Volume 129 1971 Nauk: USSR 1 228

[B23] Rieger C Skelett und muskulatur des kopfes und prothorax von Ochterus marginatus Latreille Zoomorphology 1976 83 2 109 191 10.1007/BF00993483

[B24] China WE The evolution of the water bugs Symposium on organic evolution 1955 India: Bulletin of the National Institute of Science 91 103

[B25] Mahner M Systema cryptoceratum phylogeneticum (Insecta, Heteroptera) Zoologica 1993 143

[B26] Hebsgaard MB Andersen NM Damgaard J Phylogeny of the true water bugs (Nepomorpha: Hemiptera-Heteroptera) based on 16S and 28S rDNA and morphology Syst Entomol 2004 29 4 488 508 10.1111/j.0307-6970.2004.00254.x

[B27] Li M Wang J Tian XX Xie Q Liu HX Bu WJ Phylogeny of the true water bugs (Hemiptera-Heteroptera: Nepomorpha) based on four Hox genes Entomotaxonomia 2012 34 1 35 44

[B28] Hua JM Li M Dong PZ Cui Y Xie Q Bu WJ Phylogenetic analysis of the true water bugs (Insecta: Hemiptera: Heteroptera: Nepomorpha): evidence from mitochondrial genomes BMC Evol Biol 2009 9 134 10.1186/1471-2148-9-134 19523246 PMC2711072

[B29] Stefanovic S Rice DW Palmer JD Long branch attraction, taxon sampling, and the earliest angiosperms: Amborella or monocots? BMC Evol Biol 2004 4 35 10.1186/1471-2148-4-35 15453916 PMC543456

[B30] Hillis DM Taxonomic sampling, phylogenetic accuracy, and investigator bias Syst Biol 1998 47 1 3 8 10.1080/106351598260987 12064238

[B31] Zwickl DJ Hillis DM Increased taxon sampling greatly reduces phylogenetic error Syst Biol 2002 51 4 588 598 10.1080/10635150290102339 12228001

[B32] Pollock DD Zwickl DJ McGuire JA Hillis DM Increased taxon sampling is advantageous for phylogenetic inference Syst Biol 2002 51 4 664 10.1080/10635150290102357 12228008 PMC2943957

[B33] Rannala B Huelsenbeck JP Yang Z Nielsen R Taxon sampling and the accuracy of large phylogenies Syst Biol 1998 47 4 702 710 10.1080/106351598260680 12066312

[B34] Delsuc F Scally M Madsen O Stanhope MJ de Jong WW Catzeflis FM Springer MS Douzery EJ Molecular phylogeny of living xenarthrans and the impact of character and taxon sampling on the placental tree rooting Mol Biol Evol 2002 19 10 1656 1671 10.1093/oxfordjournals.molbev.a003989 12270893

[B35] Holder M Lewis PO Phylogeny estimation: traditional and Bayesian approaches Nat Rev Genet 2003 4 4 275 284 10.1038/nrg1044 12671658

[B36] Saitoh K Sado T Mayden RL Hanzawa N Nakamura K Nishida M Miya M Mitogenomic evolution and interrelationships of the Cypriniformes (Actinopterygii: Ostariophysi): the first evidence toward resolution of higher-level relationships of the world’s largest freshwater fish clade based on 59 whole mitogenome sequences J Mol Evol 2006 63 6 826 841 10.1007/s00239-005-0293-y 17086453

[B37] Wheeler WC Schuh RT Bang R Cladistic relationships among higher groups of Heteroptera: congruence between morphological and molecular data sets Entomol Scand 1993 24 2 121 137 10.1163/187631293X00235

[B38] Graham SW Olmstead RG Barrett SC Rooting phylogenetic trees with distant outgroups: a case study from the commelinoid monocots Mol Biol Evol 2002 19 10 1769 1781 10.1093/oxfordjournals.molbev.a003999 12270903

[B39] Ware JL Litman J Klass K-D Spearman LA Relationships among the major lineages of Dictyoptera: the effect of outgroup selection on dictyopteran tree topology Syst Entomol 2008 33 3 429 450 10.1111/j.1365-3113.2008.00424.x

[B40] Lyons-Weiler J Hoelzer GA Tausch RJ Optimal outgroup analysis Biol J Linn Soc 1998 64 4 493 511 10.1111/j.1095-8312.1998.tb00346.x

[B41] Luo AR Zhang YZ Qiao HJ Shi WF Murphy RW Zhu CD Outgroup selection in tree reconstruction: a case study of the family Halictidae (Hymenoptera: Apoidea) Acta Entomologica Sinica 2010 53 2 192 201

[B42] Qiu YL Lee J Whitlock BA Bernasconi-Quadroni F Dombrovska O Was the ANITA rooting of the angiosperm phylogeny affected by long-branch attraction? Mol Biol Evol 2001 18 9 1745 1753 10.1093/oxfordjournals.molbev.a003962 11504854

[B43] Smith AB Rooting molecular trees - problems and strategies Biol J Linn Soc 1994 51 3 279 292 10.1111/j.1095-8312.1994.tb00962.x

[B44] Li T Gao CQ Cui Y Xie Q Bu W The complete mitochondrial genome of the stalk-eyed bug Chauliops fallax Scott, and the monophyly of Malcidae (Hemiptera: Heteroptera) Plos One 2013 8 2 e55381 10.1371/journal.pone.0055381 23390534 PMC3563593

[B45] Hua JM Li M Dong PZ Cui Y Xie Q Bu WJ Comparative and phylogenomic studies on the mitochondrial genomes of Pentatomomorpha (Insecta: Hemiptera: Heteroptera) BMC Genomics 2008 9 610 10.1186/1471-2164-9-610 19091056 PMC2651891

[B46] Schuh RT Slater JA True bugs of the world (Hemiptera: Heteroptera): classification and natural history: Cornell University Press 1995

[B47] Soltis DE Albert VA Savolainen V Hilu K Qiu YL Chase MW Farris JS Stefanović S Rice DW Palmer JD Soltis PS Genome-scale data, angiosperm relationships, and ‘ending incongruence’: a cautionary tale in phylogenetics Trends Plant Sci 2004 9 10 477 483 10.1016/j.tplants.2004.08.008 15465682

[B48] Delsuc F Brinkmann H Philippe H Phylogenomics and the reconstruction of the tree of life Nat Rev Genet 2005 6 5 361 375 15861208 10.1038/nrg1603

[B49] Philippe H Brinkmann H Lavrov DV Littlewood DT Manuel M Worheide G Baurain D Resolving difficult phylogenetic questions: why more sequences are not enough Plos Biol 2011 9 3 e1000602 10.1371/journal.pbio.1000602 21423652 PMC3057953

[B50] Swofford DL Olsen GJ Waddell PJ Hillis DM Hillis DM, Moritz C, Mable BK Phylogenetic inference Molecular systematics 1996 2 Sunderland, Massachusetts: Sinauer Associates 407 514

[B51] Reineke A Karlovsky P Zebitz CP Preparation and purification of DNA from insects for AFLP analysis Insect Mol Biol 1998 7 1 95 99 10.1046/j.1365-2583.1998.71048.x 9459433

[B52] Hall TA BioEdit: a user-friendly biological sequence alignment editor and analysis program for Windows 95/98/NT Nucleic Acids Symp Ser 1999 41 95 98

[B53] Lowe TM Eddy SR tRNAscan-SE: a program for improved detection of transfer RNA genes in genomic sequence Nucleic Acids Res 1997 25 5 955 964 10.1093/nar/25.5.0955 9023104 PMC146525

[B54] Tamura K Peterson D Peterson N Stecher G Nei M Kumar S MEGA5: molecular evolutionary genetics analysis using maximum likelihood, evolutionary distance, and maximum parsimony methods Mol Biol Evol 2011 28 10 2731 2739 10.1093/molbev/msr121 21546353 PMC3203626

[B55] Štys P Jansson A Check-list of recent family-group and genus-group names of Nepomorpha (Heteroptera) of the world Acta Entomol Fenn 1988 50 1 44

[B56] Thompson JD Gibson TJ Plewniak F Jeanmougin F Higgins DG The CLUSTAL_X windows interface: flexible strategies for multiple sequence alignment aided by quality analysis tools Nucleic Acids Res 1997 25 24 4876 4882 10.1093/nar/25.24.4876 9396791 PMC147148

[B57] Zhou J Liu X Stones DS Xie Q Wang G MrBayes on a graphics processing unit Bioinformatics 2011 27 9 1255 1261 10.1093/bioinformatics/btr140 21414986

[B58] Silvestro D Michalak I raxmlGUI: a graphical front-end for RAxML Org Divers Evol 2012 12 4 335 337 10.1007/s13127-011-0056-0

[B59] Posada D Crandall KA MODELTEST: testing the model of DNA substitution Bioinformatics 1998 14 9 817 818 10.1093/bioinformatics/14.9.817 9918953

[B60] McVay JD Carstens B Testing monophyly without well-supported gene trees: evidence from multi-locus nuclear data conflicts with existing taxonomy in the snake tribe Thamnophiini Mol Phylogenet Evol 2013 68 3 425 431 10.1016/j.ympev.2013.04.028 23665036

[B61] Huelsenbeck JP Hillis DM Nielsen R A likelihood-ratio test of monophyly Syst Biol 1996 45 4 546 558 10.1093/sysbio/45.4.546

[B62] Swofford DL PAUP*: Phylogenetic analysis using parsimony (* and other methods). Version 4 2003 Sunderland, Massachusetts: Sinauer Associates

[B63] Li T Hua J Wright AM Cui Y Xie Q Bu W Hillis DM Long-branch attraction and the phylogeny of true water bugs (Hemiptera: Nepomorpha) as estimated from mitochondrial genomes Dryad Digital Repository 2014 http://dx.doi.org/10.5061/dryad.tf25c 10.1186/1471-2148-14-99 PMC4101842 24884699

