# Supplementary figures and images for: Long-branch attraction and the phylogeny of true water bugs (Hemiptera: Nepomorpha) as estimated from mitochondrial genomes
Source: BMC Evol Biol. 2014 May 7;14:99. doi: 10.1186/1471-2148-14-99 (PMC4101842; doi:10.1186/1471-2148-14-99)

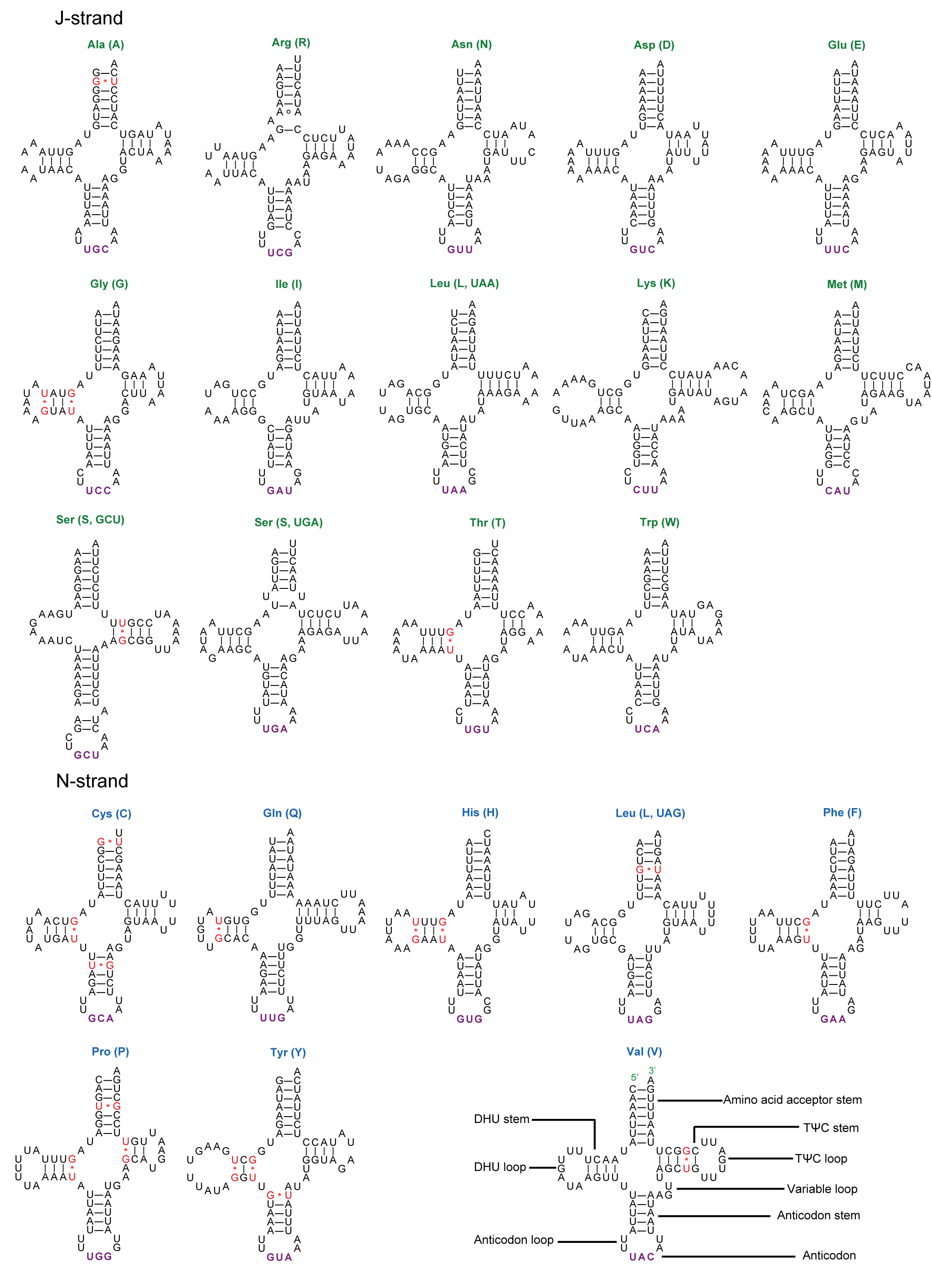

Supplement: Additional file 1 — Putative secondary structure of the 22 tRNAs identified in the mitochondrial genome of Paraplea frontalis . The tRNAs are labeled with the abbreviations of their corresponding amino acids. Dashes indicate Watson-Crick base pairing and asterisks indicate G-U base pairing. [file 1471-2148-14-99-S1.tiff]

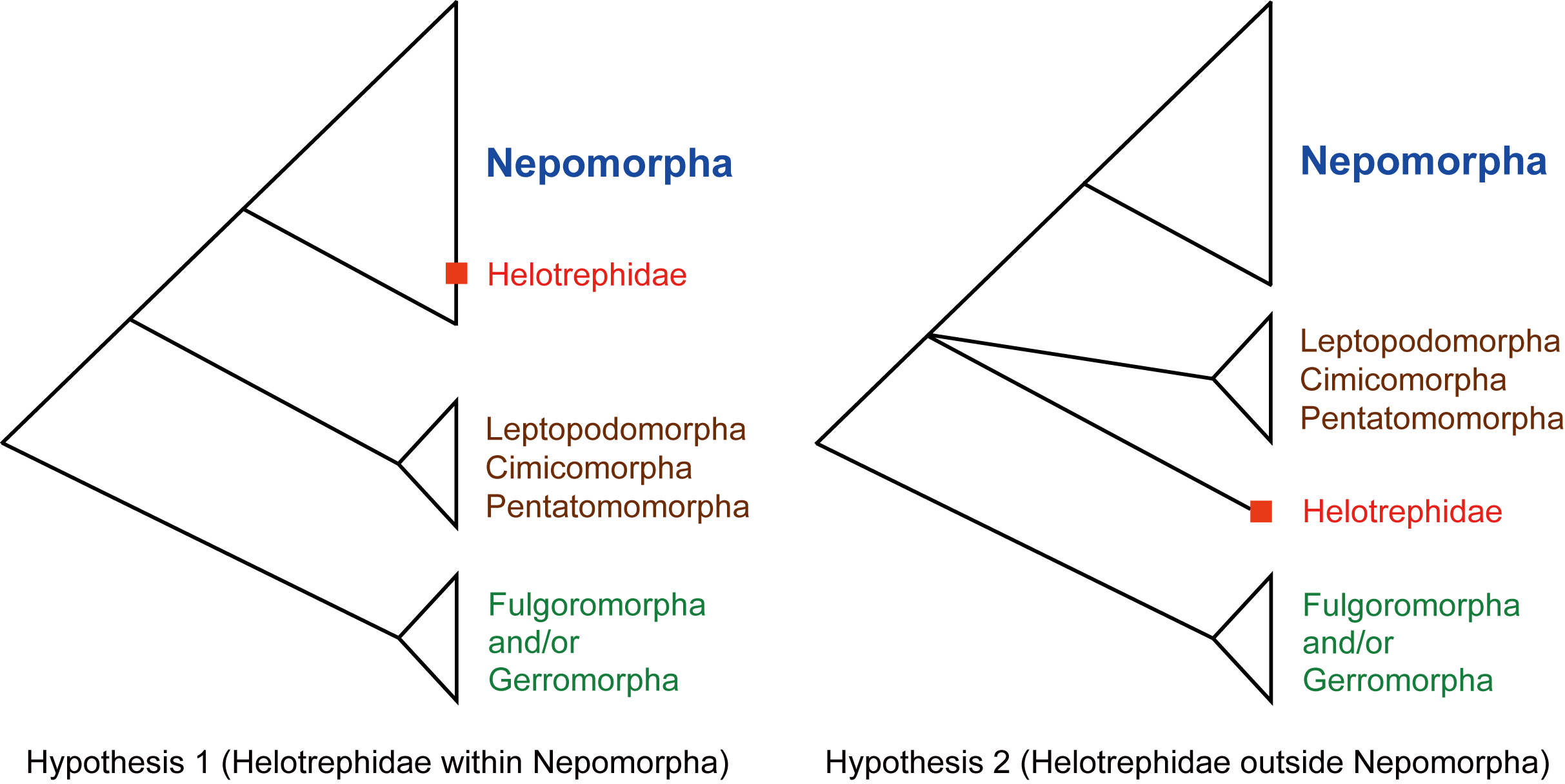

Supplement: Additional file 2 — Constraints for the two hypotheses used in the likelihood-ratio test regarding the monophyly of Nepomorpha. [file 1471-2148-14-99-S2.tiff]
