# Supplementary material for: Long-branch attraction and the phylogeny of true water bugs (Hemiptera: Nepomorpha) as estimated from mitochondrial genomes
Source: BMC Evol Biol. 2014 May 7;14:99. doi: 10.1186/1471-2148-14-99 (PMC4101842; doi:10.1186/1471-2148-14-99)
Supplement: Additional file 3 — Primers designed for Paraplea frontalis in this study. [file 1471-2148-14-99-S3.docx]

**Additional file 3 - Primers designed for *Paraplea frontalis* in this study.**

| **Primer** | **Sequence (5’-3’)** | **Binding region** |
| --- | --- | --- |
| COIF | TCTTCTACTTTATGAGCACTGGGTTTAGT | COI |
| COIR | CTCCGCTGCGTCAAGAATCATGTATTG | COI |
|  |  |  |
| CytBF | GCAACATTCCGCCATTCCACATGTTACAC | CytB |
| CytBR | AGGGGGTTAGACGAGCCAGTTTGATGTA | CytB |
|  |  |  |
| PL1F | GAGGAGGAATCTATTCTATATCACT | COI |
| PL1R | GTTTCATTTTTTCTTCTTTCTTGTC | CytB |
|  |  |  |
| PL2F | ATAAGCATGCAATTGGTGCCAACCATG | CytB |
| PL2R | AACTGTTTTGGCAGATTAGTGCGATG | COI |
