# Supplementary material for: Long-branch attraction and the phylogeny of true water bugs (Hemiptera: Nepomorpha) as estimated from mitochondrial genomes
Source: BMC Evol Biol. 2014 May 7;14:99. doi: 10.1186/1471-2148-14-99 (PMC4101842; doi:10.1186/1471-2148-14-99)
Supplement: Additional file 4 — Primers designed for Helotrephes semiglobosus semiglobosus in this study. [file 1471-2148-14-99-S4.docx]

**Additional file 4 - Primers designed for *Helotrephes semiglobosus semiglobosus* in this study.**

| **Primer** | **Sequence (5’-3’)** | **Binding region** |
| --- | --- | --- |
| HeCOIIF | ATTGGACATCAATGATATTGA | COII |
| HeND5R | TAAGGCTTTACTTTTTCTCTGTGCTGG | ND5 |
|  |  |  |
| HeND1F | TATGAAATCGTTTGAGCAACAGCAC | ND1 |
| HeCOIR | TATTTCTTGATAAAGGAGGGTAAACAGT | COI |
